# A Flexible Dual-Analyte Electrochemical Biosensor for Salivary Glucose and Lactate Detection

**DOI:** 10.3390/bios12040210

**Published:** 2022-03-31

**Authors:** Mingyang Liu, Muqun Yang, Muxue Wang, Han Wang, Jing Cheng

**Affiliations:** 1 Precision Medicine and Healthcare Research Center, Tsinghua-Berkeley Shenzhen Institute (TBSI), Tsinghua University, Shenzhen 518055, China; liumingy19@mails.tsinghua.edu.cn (M.L.); ymq14@mails.tsinghua.edu.cn (M.Y.); 2 Department of Biomedical Engineering, School of Medicine, Tsinghua University, Beijing 100084, China; wangmx21@mails.tsinghua.edu.cn (M.W.); jcheng@tsinghua.edu.cn (J.C.)

**Keywords:** electrochemical biosensing, salivary metabolites, dual-analyte biosensor

## Abstract

Electrochemical biosensors have been widely applied in the development of metabolite detection systems for disease management. However, conventional intravenous and fingertip blood tests are invasive and cannot track dynamic trends of multiple metabolites. Among various body fluids, saliva can be easily accessed and is regarded as a promising candidate for non-invasive metabolite detection. Recent works on the development of electrochemical biosensors for monitoring salivary metabolites have demonstrated high sensitivity and wide linear range. However, most of this research has been focused on salivary detection of a single metabolite. Here, we present a dual-channel electrochemical biosensor for simultaneous detection of lactate and glucose in saliva based on a flexible screen-printed electrode with two working electrodes. The sensitivities of glucose and lactate channels were 18.7 μA/(mM·cm^2^) and 21.8 μA/(mM·cm^2^), respectively. The dual-channel biosensor exhibited wide linear ranges of 0–1500 μM for the glucose channel and 0–2000 μM for the lactate channel and the cross-talk between the two detection channels was negligible, which made it adequately suitable for sensing low-level salivary metabolites. Such attractive characteristics demonstrate the potential of this dual-analyte biosensor in the development of wearable devices for monitoring disease progression and fitness.

## 1. Introduction

According to the World Health Statistics 2020, an estimated 41 million people died of chronic diseases worldwide in 2016, which constituted about 71% of all deaths. Metabolic diseases are one of the main chronic diseases, which are caused by metabolic disorders that lead to the accumulation or depletion of metabolites such as sugar, fat, electrolyte, proteins and purine [1]. Metabolites at concentrations out of the normal range not only indicate the presence of metabolic disorders but also cause severe damage to the human body. In the treatment of metabolic diseases, the detection of metabolite levels in body fluids is important. Diabetes mellitus is one of the most commonly seen metabolic diseases, which is normally caused by insulin deficiency or insulin resistance and characterized by raised blood glucose levels. For diabetes patients, the increased level of glucose in the blood may accelerate the glycolysis process of hemoglobin, which causes harm to tissues and organs all over the body. As a result, for diabetes patients, detection of blood glucose levels is of great importance not only for improving the precision of disease management but for preventing the occurrence of complications.

Metabolic disorders usually cause systematic dysfunctions and may lead to abnormal levels of a variety of metabolites [2]. As in diabetes patients, not only the blood glucose level is elevated, but also lipids, blood lipoproteins and blood lactate may exceed normal ranges because of the metabolic disorder. As a mid-product in glucose metabolisms, specifically the product of anaerobic glycolysis, lactate is closely related to glucose and both of them can largely influence each other [3]. For diabetes patients, the probability of having a lactate disorder is significant since anaerobic glycolysis will be intensified because of the elevated hemoglobin A1c level in blood. Once hypoperfusion in organs occurs because of the elevated hemoglobin A1c level, anaerobic glycolysis will aggravate and accelerate lactate production. In addition, patients who take biguanide medications or biguanide derivatives whose working mechanism is to enhance anaerobic glycolysis or cast down gluconeogenesis to reduce blood glucose levels will have a higher probability of having increased blood lactate levels [4]. Once the blood lactate concentration exceeds 13.35 mM [5], the fatality will reach 100% without noticeable signals before the crash. Therefore, the development of dual-analyte biosensing methods that can detect the dynamic trend of both lactate and glucose levels is in great demand.

Traditional methods for glucose measurement, such as colorimetry [6], chemiluminescence [7], high-performance liquid chromatography [8], and enzymatic fluorescence [9], require complicated sample pre-treatment, cumbersome instruments, and long processing time. These methods are suitable in a central laboratory or intensive care unit for their high accuracy but fail to meet the demand of decentralized medical surveillance. In recent years, electrochemical biosensing has attracted a great deal of attention in relation to monitoring the levels of metabolites owing to its advantages in terms of high sensitivity, short response time, flexibility of integration, and small footprint [10]. In this method, enzymes directly convert analyte glucose concentrations through biochemical reactions into electrical signals which can be analyzed by a compact and integrated signal acquisition and processing device. Until now, a number of commercialized products have been developed to measure blood glucose levels based on electrochemical biosensing; for example, the common home-use point-of-care devices that measure blood glucose levels use test strips with fingertip blood. However, this method is invasive and inconvenient due to the need for frequent finger pricking. In recent years, patch-like continuous glucose monitoring (CGM) systems that measure the dynamic trend of glucose levels in interstitial fluids have been introduced [11,12,13]. However, these CGM devices are designed for detection of a single metabolite alone and cannot meet the need for simultaneous multi-metabolite detection. In addition, significant effort has been made towards the development of non-invasive biosensors for metabolite monitoring.

Body fluids, such as saliva, tears, sweat and urine, have been utilized as they can be sampled in a non-invasive manner. These body fluids can be readily accessed without disrupting the protecting layers of the skin [14]. Moreover, access to these body fluids does not rely on microneedles, as in the case of interstitial fluids, greatly simplifying system design and application. Of these fluids, saliva has been considered extremely promising for such non-invasive monitoring. Plenty of constituents in the blood are permeable to saliva via transcellular or paracellular paths; as a result, good correlations between concentrations of numerous analytes in blood and saliva have been established [15,16]. The unique properties of saliva, such as easy accessibility and the presence of plentiful biomarkers, make it particularly attractive for health surveillance and monitoring. Recent progress in point-of-care and wearable biosensors has led to the development of non-invasive glucose detection methods. Wang et al. presented a mouthguard biosensor modified with Prussian blue and lactate oxidase for detection of salivary lactate [17]. Later, they presented an integrated system for salivary urine detection [18]. Arakawa et al. developed a screen-printed electrode for salivary glucose detection [19]. However, in these studies only a single metabolite has been detected and analyzed.

Recently, multi-metabolite detection has drawn a significant amount of attention with the advancement of biosensing technology. Kim et al. developed a tattoo-based electrochemical biosensor that can detect alcohol and glucose in sweat and interstitial fluid, respectively [20]. They integrated eight electrodes in the sensing module: three electrodes for glucose detection, three electrodes for lactate detection and one pair of electrodes for iontophoresis. In another work by Zhang et al., three-electrode biosensors for glucose, lactate, ascorbic acid, uric acid, Na^+^, and K^+^ have all been integrated in a one-piece wearable device, and fifteen electrodes in total were used [21]. A great number of electrodes will make the complete device significantly large in terms of its footprint and the peripheral circuit design will be complicated for signal processing. Although these works can achieve multi-metabolite detection, the systems are complicated, however, and have large footprints.

Here, we report the development of a flexible dual-analyte electrochemical biosensor for simultaneous detection of glucose and lactate in saliva. Flexible polyethylene glycol terephthalate (PET) was used as the substrate for electrode fabrication by screen printing. Ferrocene was utilized as the electron mediator to achieve molecular wiring of the enzymes to the electrode in this work, avoiding cross-talk between the two working electrodes and latent signal interference caused by hydrogen peroxide. The dual-analyte biosensor showed great performance even under low bias potential, and the linear ranges of glucose and lactate measurement of the biosensor corresponded well with physiological levels of these metabolites in saliva. The sensitivity of the dual-analyte biosensor for glucose detection was 18.7 μA/(mM·cm^2^) and 21.8 μA/(mM·cm^2^) for lactate detection. Overall, the proposed biosensor facilitates multiplexed detection of different analytes in a single electrolytic cell by sharing the reference and counter electrodes. A timing modulation data acquisition method was adopted for the detection of response signals of multiple analytes.

## 2. Materials and Methods

### 2.1. Dual-Analyte Biosensor Design

**Figure 1:**
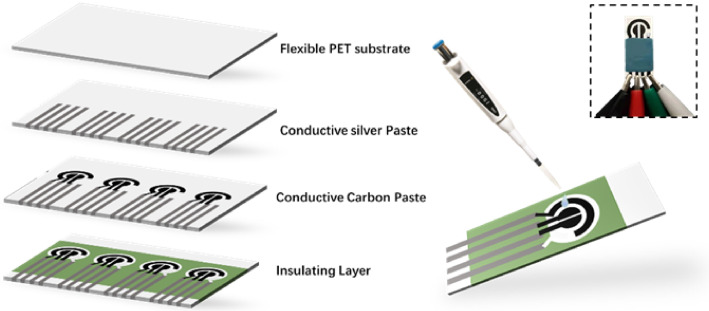
Schematic diagram of the fabrication process of the dual-analyte biosensor. The inset shows the photograph of the dual-analyte biosensor.

For electrochemical biosensing of metabolites, the three-electrode system is the most commonly used. It consists of a working electrode, a counter electrode, and a reference electrode. In order to avoid high overvoltage and decrease the current density, the size of the counter electrode is normally designed to be large. As illustrated in Figure 1, in our design, the counter electrode was made of conductive carbon paste and was in the outermost part of the sensing area. To reduce the size of the whole sensing area, two working electrodes were patterned in the middle of the sensing area and shared a pair of counter electrode and reference electrode. The electrodes were screen-printed on a flexible PET substrate. In the case of dual-metabolite detection, using this kind of three-electrode system, the potential diffusion of hydrogen peroxide in the analyte solution between the two closely placed working electrodes may cause signal interference. To solve this problem, the electron transfer mediator was employed in the process of enzyme immobilization. By using ferrocene as the electron mediator, molecular wiring of the enzymes to the electrodes was achieved, thus lowering the working potential of the electrodes and avoiding cross-talk between the two closely placed working electrodes and signal interference caused by hydrogen peroxide. Dual-metabolite detection was thereby realized using only four electrodes in a tightly packed electrode pair in our design.

### 2.2. Reagents and Equipment

Glucose oxidase (GOD, EC 1.1.3.4), L-lactate sodium, glucose, and ferrocene were purchased from Sigma-Aldrich (St. Louis, MO, USA) and used without further purification. Urea, ≥99.5% (T) and L-ascorbic acid were purchased from Aladdin (Shanghai, China). Lactate oxidase (LOD, ≥80 units/mg solid) was purchased from Yuanye Bio-Technology Co., Ltd. (Shanghai, China). The Ag conductive paste and carbon paste were purchased from the Ausbond Corporation (Silver paste 3813 and carbon paste A528, Ausbond Corporation, Shenzhen, China). Bovine serum albumin (BSA) and phosphate buffer solution (PBS, pH 7.4) were purchased from Solarbio (Beijing, China). Glutaraldehyde solution (50%) was obtained from TCI (Shanghai, China), and PET was purchased from Shenzhen Shengjia Materials Company (Shenzhen, China). All other chemicals used were of analytical grade. Double-distilled water was used throughout the experiments. All of the electrochemical measurements were performed using the CHI760E electrochemical workstation (Chenhua, Shanghai, China).

### 2.3. Screen-Printed Electrode Fabrication

Before printing, the PET substrate was washed with ethanol for 10 min in the ultrasonic tank. The first layer of carbon paste was deposited to make the working electrode and the counter electrode by screen printing. The printed electrodes were then annealed at 115 °C for 15 min. The second layer of the reference electrode was printed with Ag/AgCl paste and then cured at 115 °C for 10 min. Then, silver paste was deposited by screen printing for electrical connections. Finally, the cover layer was printed with insulating polymer paste. The screen-printed electrodes were stored at room temperature before further modification.

### 2.4. Modification of the Working Electrodes

In the modification of the working electrodes, the electron mediator ferrocene was utilized to minimize cross-talk. First, 5 mg of ferrocene was dissolved in 10 mL ethanol. Then, 5 μL of this solution was pipetted onto the surface of the two working electrodes, respectively. The solution was allowed to dry at room temperature.

Enzymes were immobilized on the working electrodes as follows. For modification of the GOD working electrode, a mixture of 8 mg of GOD and 6 mg of BSA was prepared and dissolved in 200 μL of 0.01 M phosphate buffer (pH 7.4). Then, 20 μL of glutaraldehyde at a concentration of 2.5% was added into the mixture solution and mixed thoroughly using a vortex mixer. Next, 6 μL of the above solution was immediately pipetted onto the surface of the working electrode and allowed to dry overnight at 4 °C. Afterwards, the enzyme mixture layer was covered by a thin layer of Nafion by applying 3 μL of Nafion solution at a concentration of 0.25% and allowed to dry. For modification of the LOD working electrode, the procedures were the same, except the components of the enzyme mixture were changed to 1 mg of LOD and 10 mg of BSA. Finally, the modified dual-analyte biosensor was stored at 4 °C until use.

### 2.5. Electroanalytical Performance Characterization

The electrochemical performance of the dual-analyte biosensor was evaluated by dissolving different concentrations of lactate and glucose in 0.01 M phosphate buffer (pH 7.4). Glucose was spiked in PBS (1×, pH = 7.4) to prepare solutions with the concentrations ranging from 0−1000 μM, and lactate solution was prepared in the same way with the concentrations ranging from 0−1.75 mM. Glucose or lactate solutions (200 μL) were dropped onto the detection chamber of the biosensor by pipetting. Amperometric I-t tests were carried out to measure the signal response of lactate solutions by stepping the potential to 0.2 V versus the reference electrode, while the measurements of glucose were carried out at a bias potential of 0.25 V. The electrochemical signals were recorded for 30 s.

### 2.6. Interference Study

The interference test was performed using the dual-channel mode of the CHI760E electrochemical station. Urea and ascorbic acid are common interfering substances present in body fluids and were chosen as the interference testing substances. The concentrations of urea and ascorbic acid were determined according to physiological concentrations in saliva, with urea at a concentration of 4 mM and ascorbic acid at a concentration of 10 µM, respectively. The stock solutions of glucose, lactate, urea, and ascorbic acid were prepared in PBS at concentrations of 1 M, 0.15 M, 1 M, and 0.15 M, respectively. During the test, the bias potential of the working electrode for lactate was set be 0.2 V, while that of the working electrode for glucose was set to be 0.25 V. Stock solutions with interfering substances and target analytes were added into the electrolytic cell in series, and the current responses from both channels were recorded.

The experimental data were processed with Origin 2019b. The neighborhood averaging method was used as the filtration method to reduce the noise.

### 2.7. Human Saliva Test

Human saliva samples were collected from healthy volunteers under fasting conditions (fasting for at least 8 h). The collected saliva samples were centrifuged at 8000 rpm at 4 °C for 8 min. The precipitants were removed and the supernatants were collected and stored at −20 °C before use. Prior to testing, the samples were spiked with stock solutions to pre-set concentrations. Due to the high viscosity of the undiluted saliva samples, the saliva samples and stock solutions were blended using a vortex mixer after spiking. The stock solutions were prepared by dissolving lactate and glucose separately in PBS medium at a concentration of 50 mM. Glucose stock solutions with the volumes of 1 μL, 2 μL, 3 μL, 4 μL, and 5 μL were added into human saliva aliquots to make 250 μL of spiked glucose samples at the final concentrations listed in Table 1. Spiked lactate solutions were prepared in the same manner by adding lactate stock solutions with the volumes of 2.5 μL, 3 μL, 3.5 μL, 4 μL, 4.5 μL, and 5 μL to human saliva aliquots to make 250 μL of spiked lactate samples at the final concentrations listed in Table 1. During the test, 200 μL of mixed analyte solutions were pipetted from the aliquots to the detection chamber and amperometric I-t measurements were performed. The current response of both glucose and lactate measurements were recorded continuously for 40 s.

## 3. Results and Discussion

### 3.1. Electrochemical Characterization

**Figure 2:**
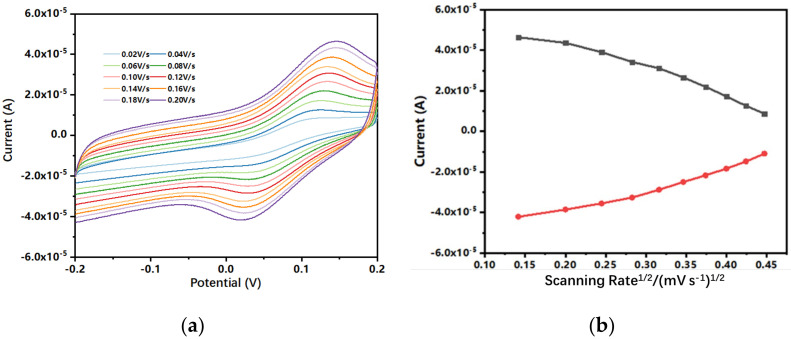
(**a**) Cyclic voltammograms of 0.5 mM ferrocene carboxylic acid in 0.1 M KCl (containing 5 mM [Fe(CN)_6_]^3-/4-^) at different scanning rates: 20, 40, 60, 80, 100, 120, 140, 160, 180, and 200 mV/s. (**b**) Calibration plots of the anodic (I_pa_) and cathodic peak current (I_pc_) versus the square of the scanning rate.

Figure 2a shows the cyclic voltammograms of the biosensor under different scanning rates. The oxidation current increased as the scanning rate increased from 20 to 200 mV/s with 20 mV/s interval. Figure 2b shows that the peak oxidation current of all cyclic voltammetry curves increased in a linear manner as the scanning rate increased. The redox peak of the CV curves exhibited a typical reversible electrochemical reaction pattern in which the rate of reaction was governed by the diffusion of the electroactive species to the electrode surface. The separation between the peak cathodic potential and the peak anodic potential was 120 mV. In addition, the position of peak potentials did not alter as a function of the potential scanning rates, and the anodic peak current was approximately equal to the cathodic peak current in the range of 20 to 200 mV/s. These results suggest that the kinetics of electron transfer is sufficiently rapid to maintain the surface concentrations of redox active species at the values required by the Nernst equation.

The electrochemical impedance spectroscopy was also adopted to evaluate the electron transfer performance of the screen-printed electrodes. Using the [Fe(CN)_6_]^4-/3-^ couple as redox probe, the electron transfer resistance (R_ct_) of the two working electrodes was determined.

**Figure 3:**
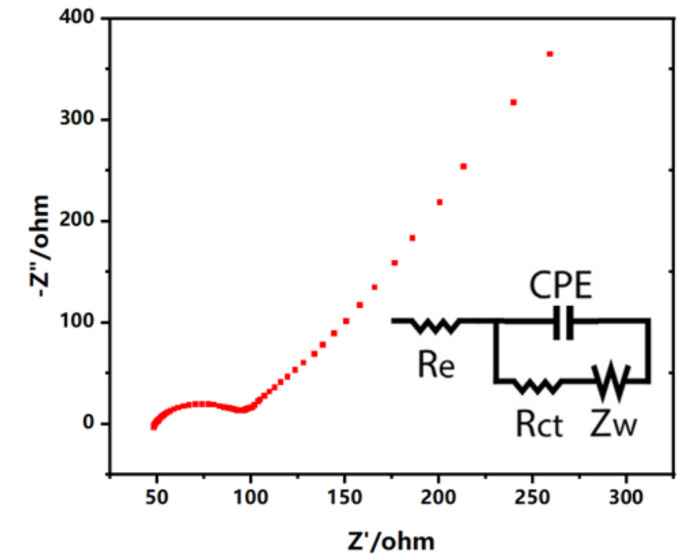
EIS spectra of the screen-printed electrodes obtained in 0.1 M KCl (containing 5 mM [Fe(CN)_6_]^3-/4-^). The insert is the Randles equivalent circuit of the biosensor.

Fitting of the spectra was performed using the equivalent electrical circuit shown in Figure 3, which consists of the electrolyte resistance (R_e_) in series with a parallel combination of the interfacial charge transfer resistance (R_ct_), the Warburg impedance (Z_w_) that stands for the diffusion of the analytes in solution, and a capacitance element (CPE). The R_ct_ of the working electrode was characterized impedance (Z_w_) that stands for the diffusion of the analytes in solution and a capacitance element (CPE). The R_ct_ of the working electrode was characterized to be equal to 50.2 Ω. The low R_ct_ value of the electron transfer resistance shows the high efficiency of electron transfer of the electrochemical probe (ferro/ferricyanide) using the fabricated dual-analyte biosensor.

### 3.2. Sensitivity Test

Amperometric I-t tests with bias potentials of 0.25 V for glucose and 0.2 V for lactate were performed to measure the current signal responses when the screen-printed electrode was immersed in either lactate or glucose solutions at different concentrations, respectively.

**Figure 4:**
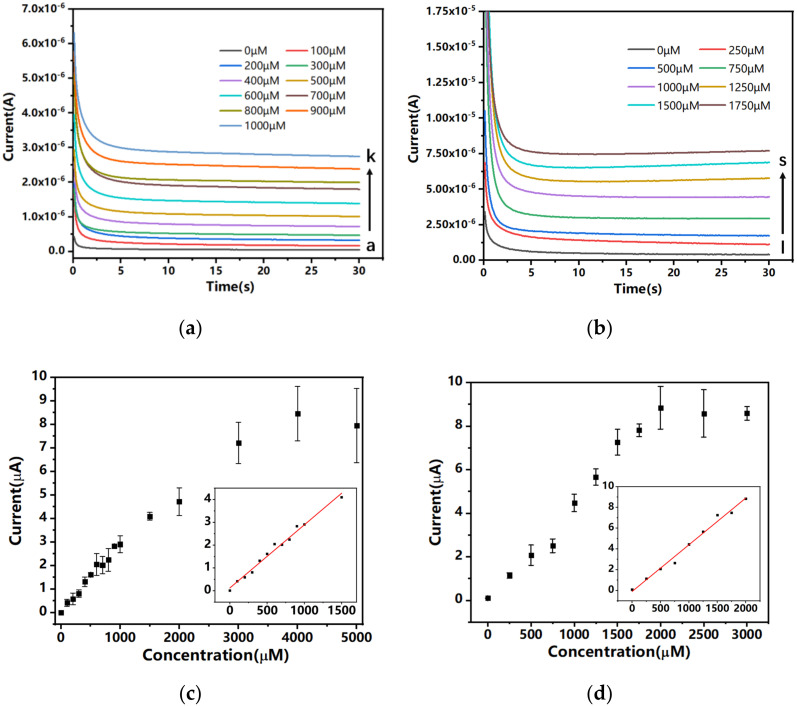
Amperometric I-t curves of the dual-channel biosensor in different concentrations of (**a**) glucose and (**b**) lactate solutions. (**c**) Steady-state currents plotted as functions of glucose concentration. The insert shows the linear curve fitting between response currents and glucose concentrations, coefficient of association R^2^ = 0.9860 (*N* = 3). (**d**) Steady-state currents plotted as functions of lactate concentration. The insert shows the linear curve fitting between response currents and lactate concentrations, coefficient of association R^2^ = 0.9876 (*N* = 3).

The results from Figure 4 indicate that the dual-analyte biosensor possessed a wide linear range and high sensitivity in glucose and lactate detection. As shown in Figure 4a, the response current increased gradually as the concentration of glucose increased from 0 μM to 1000 μM. A similar phenomenon was observed for lactate detection in Figure 4b, with a linear range of 0 μM to 1.5 mM. As calculated, the sensitivity of glucose detection in PBS medium was 18.7 μA/(mM·cm^2^), while for lactate detection the sensitivity was 21.8 μA/(mM·cm^2^).

### 3.3. Interference Study

Whether other electroactive substances in saliva would interfere with the signal response of the electrochemical biosensor was to be considered in the experiment. The interference test was performed using the dual-channel mode of the CHI760E electrochemical station. In this mode, the electrochemical station can control the potentials of the two working electrodes in one electrolytic cell at the same time. The dual-analyte biosensor was immersed in PBS medium which was spiked with target analytes, such as glucose and lactate, as well as interfering substances, such as uric acid and ascorbic acid.

**Figure 5:**
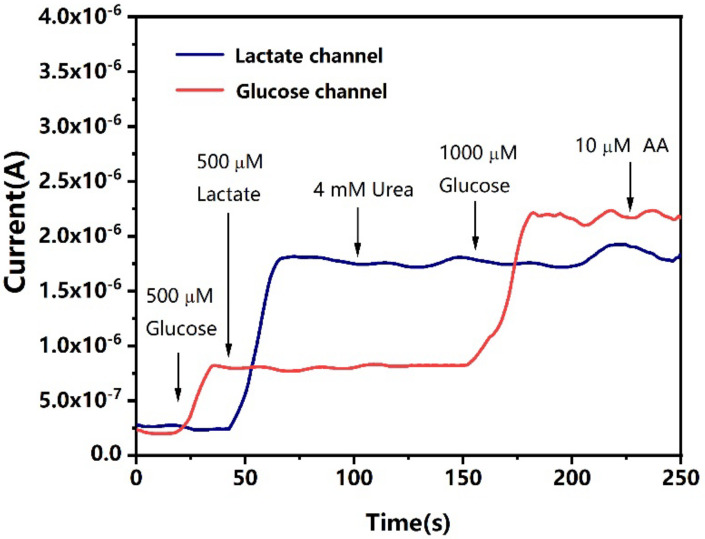
Current response from the two channels of the electrochemical biosensor. The arrows show the time points at which the analytes (glucose and lactate) and interfering substances were added into the electrolytic cell. Current response measured by amperometric I-t test was obtained.

An amperometric I-t test was performed and the signal response from the two channels were recorded at the same time (Figure 5). Twenty seconds after the test started, 15 μL of the glucose stock solution was added to the electrolytic cell to make a total of 30 mL glucose solution at the final concentration of 500 μM. The current response of the glucose channel increased instantly, while the current of the lactate channel remained at low levels. In another 25 s, 100 μL of the lactate stock solution was added into the electrolytic cell to make the final concentration of lactate 500 µM. The current response from the lactate channel increased while the current of the glucose channel was unaffected. When a high concentration of glucose at 1000 μM final concentration was added to the electrolytic cell at 150 s, again, only the glucose channel observed current response change. These results show that although the working electrodes of the dual-analyte biosensor shared a common counter electrode and reference electrode, the signal responses did not interfere with each other. Furthermore, we added 4 mM final concentration of urea and 10 μM final concentration of ascorbic acid into the electrolytic cell at the time points of 100 s and 220 s, respectively, and no significant change in current response in either the glucose channel or the lactate channel was observed. Therefore, good specificity of the dual-analyte biosensor was validated, and no cross-talk between the two detection channels was observed.

### 3.4. Human Saliva Test

**Figure 6:**
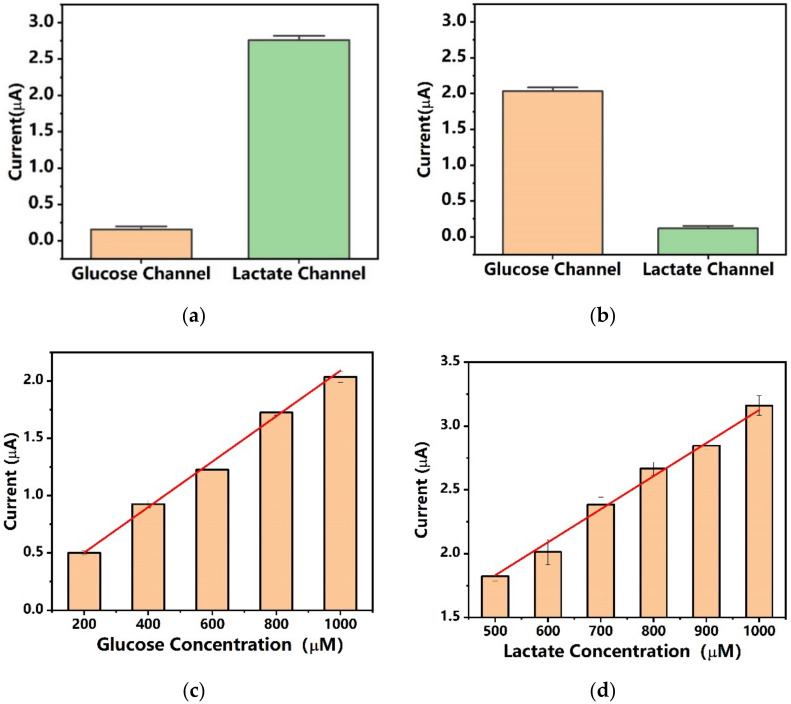
The current response from the two channels of the dual-analyte biosensor with spiked human saliva samples. (**a**) The measured current of the dual-analyte biosensor using human saliva sample 11 spiked with 0 µM glucose and 1000 µM lactate (*N* = 3). (**b**) The measured current of the dual-analyte biosensor using human saliva sample 5 spiked with 1000 µM glucose and 0 µM lactate (*N* = 3). (**c**) The measured current of the glucose detection channel using human saliva samples 1, 2, 3, 4, and 5 spiked with 200, 400, 600, 800, and 1000 µM glucose (*N* = 3). (**d**) The measured current of the lactate detection channel using human saliva samples 6, 7, 8, 9, 10, and 11 spiked with 500, 600, 700, 800, 900, and 1000 µM lactate (*N* = 3).

After evaluation of the dual-analyte biosensor in the synthetic buffer matrix, experiments were carried out using human saliva samples. The current response of the sensor to different glucose and lactate levels was examined using unstimulated human saliva from fasting subjects spiked with different concentrations of analytes. The glucose and lactate analytes in spiked human saliva samples adopted the same concentrations as in Table 1. Another interesting question was whether the signal responses of the two working electrodes would affect each other during the test. As shown in Figure 6a,b, the electrochemical process that occurred on the surface of the biosensor had a negligible effect on the response signal of the other channel.

The amperometric response of human saliva samples spiked with 200, 400, 600, 800, and 1000 µM glucose was measured with 0.25 V bias potential. The dual-analyte biosensor demonstrated excellent performance with high sensitivity and high linearity. The sensitivity of the dual-analyte biosensor in glucose measurement using spiked human saliva was 7.9 μA/(mM·cm^2^). As estimated, the endogenous glucose level of the test subject was 53.8 μM, which was in the normal range of saliva glucose levels of healthy individuals. As a reference, for non-diabatic healthy individuals the glucose level in saliva is normally less than 120 µM, while for diabatic patients the glucose level in saliva can reach 1 mM. For the measurement of lactate, human saliva was spiked with 500, 600, 700, 800, 900, and 1000 μM lactate. The results showed that the sensitivity of the dual-analyte biosensor in lactate measurement using spiked human saliva was 11.9 μA/(mM·cm^2^), and the endogenous lactate level of the test subject was estimated to be 206 μM, which was in the normal range of saliva lactate of healthy individuals at rest without stimulation. As a reference, the normal range of lactate in the saliva of individuals is 0.1–2.5 mM. Compared to the measurement in buffer media, the current increment due to the increase in glucose and lactate concentrations was smaller, which may be attributed to the high viscosity of the saliva liquid. The results show that the dual-analyte biosensor has good performance in real saliva sample measurement.

## 4. Conclusions

In this work, we have developed a dual-analyte biosensor for simultaneous detection of glucose and lactate in saliva. The bare electrodes were fabricated with screen-printing techniques on a flexible PET substrate and then modified with a ferrocene and BSA/enzyme complex. Ferrocene was used as the electron mediator to transfer redox electrons from enzymes to the working electrodes to avoid the possible signal cross-talk generated from the diffusion of dissolved active species. As a result, negligible cross-talk between the two working electrodes was observed during electrochemical measurements. The fabricated biosensor showed a good sensitivity of 18.7 μA/(mM·cm^2^) for the glucose channel and 21.8 μA/(mM·cm^2^) for the lactate channel. It also showed good linearity fittings of R^2^ = 0.98 in physiological ranges of glucose and lactate in both PBS and saliva solutions. Moreover, although sharing one electrolytic cell and the same reference and counter electrodes, the signal responses from the two different channels showed no cross-talk with each other. The two working electrodes exhibited good response to their target analytes. The dual-analyte biosensor can help monitor and provide alerts of abnormal glucose and lactate concentrations in saliva and thus be a powerful tool for disease management and prediction. In future studies, the developed dual-analyte biosensor could be integrated in wearable dental devices to carry out continuous monitoring of metabolite levels in patients. Moreover, three-channel or multi-channel biosensors could be developed based on the same principle and provide actionable information about multiple metabolites and generate comprehensive information of an individual’s wellness.

Overall, the developed biosensor enables high-sensitivity, high-specificity and high-stability electrochemical detection of multiple metabolites in saliva samples. Such attractive performance substantiates the potential of the dual-analyte biosensor as a practical approach for continuous non-invasive monitoring of physiological conditions of disease progression and health status.

## Figures and Tables

**Table 1:** The spiking concentrations of the tested human saliva samples.

Sample Number	Final Glucose Concentration (µM)	Final Lactate Concentration (µM)
1	200	0
2	400	0
3	600	0
4	800	0
5	1000	0
6	0	500
7	0	600
8	0	700
9	0	800
10	0	900
11	0	1000
